# Postoperative management with dexmedetomidine in a pregnant patient who underwent AVM nidus removal: a case report

**DOI:** 10.1186/s40981-017-0085-6

**Published:** 2017-04-24

**Authors:** Chanatthee Kitsiripant, Kotoe Kamata, Rie Kanamori, Koji Yamaguchi, Makoto Ozaki, Minoru Nomura

**Affiliations:** 1 Department of Anesthesiology, School of Medicine, Tokyo Women’s Medical University, 8-1 Kawada-cho Shinjuku-ku, Tokyo, 1628666 Japan; 2 Department of Anesthesiology, Faculty of Medicine, Prince of Songkla University, 15 Karnjanavanich Road, Hat Yai, Songkhla 90110 Thailand; 3 Department of Neurosurgery, School of Medicine, Tokyo Women’s Medical University, 8-1 Kawada-cho Shinjuku-ku, Tokyo, 1628666 Japan

**Keywords:** Intracranial arteriovenous malformation, Pregnancy, Normal perfusion pressure breakthrough, Dexmedetomidine

## Abstract

**Background:**

Following cerebral arteriovenous malformation (AVM) surgery, severe brain edema and hemorrhage may be caused by postoperative normal perfusion pressure breakthrough (NPPB). Sedation is necessary for this population. It is a challenge for the anesthesiologist to maintain hemodynamic stability without interfering with the neurological assessment. In Japan, propofol is contraindicated for pregnant patients. Dexmedetomidine is a versatile drug in anesthesia practice and may be useful for this situation. There is no report using dexmedetomidine for the purpose of NPPB control in pregnant patients. We describe the postoperative management with dexmedetomidine for a pregnant patient who underwent cerebral AVM nidus removal.

**Case presentation:**

A 32-year-old patient presented with headache at the 16th week of gestation. Neuroimaging revealed an intraventricular hemorrhage and an AVM at the right anterior horn of the lateral ventricle which caused bleeding. A multidisciplinary team discussion was done, and then a craniotomy for AVM nidus removal was performed under general anesthesia. Preanesthetic aspiration prophylaxis and rapid sequence induction were added to our conventional anesthetic management. Hypotension occurred after anesthetic induction but the patient recovered by volume resuscitation and vasopressors. Anesthesia was maintained with 50% O_2_ in air and sevoflurane. The AVM was completely removed, and no perioperative complications occurred. Postoperative sedation with dexmedetomidine was used to prevent breakthrough hyperperfusion and cerebral edema.

**Conclusions:**

Dexmedetomidine infusion was used for postoperative sedation without causing any side effects, and it can be an alternative for sedation, especially when propofol is contraindicated.

## Background

Neurosurgical intervention during pregnancy is still a challenge due to the strict surgical indications and maternal-fetal effects of anesthetic drugs. Arteriovenous malformation (AVM) accounts for about 50% of subarachnoid hemorrhage in pregnant women [1]. It has been considered that hormonal changes to the arterial wall and increased cardiac output are related to the increased risk of AVM rupture, especially in the second and the third trimester. The risk of rebleeding is 25% during the same pregnancy compared with 3–6% risk during the first year in nonpregnant women [2]. Neurosurgical resection represents the definitive treatment because it eliminates rebleeding [2–4]. In addition, after excision of an AVM, normal perfusion pressure breakthrough (NPPB) impairs autoregulation which causes severe neurological damage and should be prevented by postoperative sedation. There are many medication options for sedation such as propofol, benzodiazepines, or dexmedetomidine. However, the maternal use of propofol is prohibited in Japan. Dexmedetomidine is a versatile agent in anesthesia practice and can be used as an alternative in this circumstance. It induces conscious sedation/analgesia while preserving respiratory and cardiovascular functions with cerebral hemodynamic stabilizing properties. Nowadays, the existing literature on the use of dexmedetomidine in obstetric patients for neurosurgery is limited. The purpose of this report is to describe our experience in postoperative management with dexmedetomidine for a pregnant patient who underwent cerebral AVM nidus removal.

## Case presentation

A 32-year-old secundigravida presented with constant headache and complained of disorientation in her day-to-day life at 16 weeks of gestation. Because her headache was not controlled with 400 mg of acetaminophen, she visited a nearby hospital at week 17 of gestation. She was conscious and alert. She was not hypertensive; her heart rate (HR) was 83 beats/min and her blood pressure (BP) was 103/50 mmHg. Obstetric ultrasonography showed a single viable fetus without any fetal compromise. Computed tomography (CT) and magnetic resonance imaging revealed intraventricular hemorrhage, but there was no evidence of increased intracranial pressure. Three-dimensional contrast-enhanced computed tomography angiography showed a 1.5 cm AVM nidus fed from a branch of the right Heubner’s artery and intraventricular venous drainage. At week 18 of gestation, she was referred to our institution for further treatment. Her AVM was classified as grade 2 in the Spetzler-Martin grading system. After a preoperative multidisciplinary conference including neurosurgeons, obstetricians, and anesthesiologists, AVM nidus removal was planned at week 21 of gestational age under general anesthesia.

The patient was premedicated with famotidine 20 mg intravenously. Standard cardiorespiratory monitoring, invasive BP, and bispectral index (BIS) monitoring were adopted. Left uterine displacement was done, and the fetal heart rate (FHR) was reassured before the induction of anesthesia. After preoxygenation, rapid sequence induction with cricoid pressure was performed with fentanyl 150 mcg, thiopental 250 mg, and rocuronium 50 mg. Intubation was successfully done on the first attempt with a McGRATH™ MAC video laryngoscope. Maternal BP decreased to 83 mmHg after the anesthetic induction, but it subsequently recovered with volume resuscitation and vasopressors that included ephedrine 4 mg and two boluses of phenylephrine 0.05 mg. Anesthesia was maintained with 50% oxygen in air, sevoflurane, and remifentanil 0.3–0.5 mcg/kg/min. End-tidal concentration of sevoflurane was titrated according to the depth of anesthesia. During the operation, the systolic BP was maintained in the range of 90–120 mmHg, HR of 82–86 beats/min, peripheral oxygen saturation of 99–100%, an end-tidal CO_2_ of 33–40 mmHg, and BIS value of 40–60. The AVM was completely and successfully removed. Estimated blood loss was 50 mL, and 850 mL of bicarbonate Ringer’s solution and 1000 mL of hydroxyethyl starch were infused. The total duration of the surgery and anesthesia was 186 and 259 min, respectively. FHR monitoring confirmed fetal viability at the end of anesthesia. In consequence, dexmedetomidine 1 mcg/kg was administered over 10 min as a loading dose then followed by a maintenance infusion of 0.7 mcg/kg/h. After the dexmedetomidine infusion was initiated, her vital signs were stable without hypotension or bradycardia. Complete resection of the AVM without intraventricular hemorrhage and cerebral ischemia were confirmed by postoperative CT. In the intensive care unit (ICU), she was awake while the maintenance infusion rate of dexmedetomidine was 0.35 mcg/kg/h. One hour after surgery, the patient recovered fully awake without neurological deficit. Dexmedetomidine infusion was then stopped, and extubation was performed. The total dexmedetomidine dose used was 99.5 mcg, and the duration of infusion was 2 h. Multimodal analgesia of intravenous fentanyl and acetaminophen was effective to control postcraniotomy headache without postoperative nausea and vomiting. Her analgesic requirement was reduced, and her postoperative pain score was 0. She was discharged from the ICU on postoperative day 1 and went home on postoperative day 10. There were no obstetric complications, and the pregnancy proceeded uneventfully.

### Discussion

After an excision of a high-flow AVM, there is the potential risk for postoperative neurological complications due to NPPB which leads to hyperemia, cerebral edema, and intracranial hemorrhage without evidence of residual AVM [5]. Patients at risk for NPPB may benefit from blood pressure control and sedation. The challenge for the anesthesiologist is to select a technique that minimizes perioperative hemodynamic instability, provides analgesia, and diminishes the risk of postoperative complications. Our case report showed that intraoperative loading of dexmedetomidine provided smooth transition from intraoperative to postoperative patient care. It contributed to a smooth recovery without cardiovascular instability and had the advantage of postoperative neurological assessment due to conscious sedation.

In postoperative sedation of obstetric patients, no sedative drug has been reported as the drug of choice due to a lack of adequate or well-controlled studies. Propofol may be associated with lower initial Apgar scores at the time of cesarean section and may cause mild metabolic acidosis during a long neurosurgical procedure in pregnant patients. Moreover, in several countries, including Japan, propofol is stated by the manufacturer to be contraindicated during pregnancy [3, 6]. Benzodiazepines present the risk of congenital malformations, and central nervous system abnormalities, respiratory depression, floppy infant syndrome, and potential neonatal withdrawal [7, 8].

Dexmedetomidine is a highly selective alpha-2 adrenergic agonist which is a useful agent because it provides anxiolytic and sympatholytic effects, analgesia, and conscious sedation without respiratory depression. The existing literature reports on dexmedetomidine use in pregnant patients who underwent neurosurgery are limited. Recently, Handlogten and colleagues revealed that dexmedetomidine infusion allowed adequate sedation for an awake neurosurgical procedure in a pregnant patient without causing any adverse effects [9]. For pregnant patients, several studies mentioned that dexmedetomidine was used safely in many interventions such as sedation during noninvasive ventilation [10], as an alternative for maintenance of general anesthesia [9, 11], blunt hemodynamic response to intubation, and maintained hemodynamic stability without neonatal respiratory depression during cesarean section under general anesthesia [12, 13]. However, it can cross the placenta to the fetus [12, 14]. Dexmedetomidine use is recommended only if the benefit is clearly needed and the benefits overcome the risks to the fetus. In neurosurgery, dexmedetomidine decreases cerebral blood flow that could be the result of a decrease in cerebral metabolic activity [15, 16]. The Acute Neurosurgical ICU Sedation Trial reported that sedation with dexmedetomidine improved the cognitive score compared with propofol [17]. For postoperative care, a meta-analysis by Jin and colleagues found that continuous infusion of dexmedetomidine had the advantage of preventing postoperative nausea and vomiting and could reduce potential adverse events such as bradycardia and hypotension [18]. Su and colleagues studied the opioid-sparing effect of perioperative dexmedetomidine plus sufentanil infusion during neurosurgery and reported that dexmedetomidine (0.02 mcg/kg/h) plus sufentanil (0.02 mcg/kg/h) could reduce postoperative opioid consumption and improve pain scores [19]. Besides, dexmedetomidine was associated with a notably lower incidence of postoperative delirium [20, 21].

## Conclusions

Dexmedetomidine is considered a multipurpose agent for pregnant patients who undergo neurosurgery. It is a sedative agent with minimal effects on respiratory function, maintains hemodynamic stability and cerebral protection with analgesic effects, and also decreases the risk of postoperative complications. Because propofol is prohibited for maternal use in Japan, our case showed that dexmedetomidine is an alternative to propofol for sedation in the obstetric population.

## Abbreviations

AVM: Arteriovenous malformation
BIS: Bispectral index
BP: Blood pressure
CT: Computed tomography
FHR: Fetal heart rate
ICU: Intensive care unit
NPPB: Normal perfusion pressure breakthrough
